# NAV-KIDS^2^ trial: protocol for a multi-centre, staggered randomised controlled trial of a patient navigator intervention in children with chronic kidney disease

**DOI:** 10.1186/s12882-019-1325-y

**Published:** 2019-04-18

**Authors:** Anita van Zwieten, Patrina Caldwell, Kirsten Howard, Allison Tong, Jonathan C. Craig, Stephen Alexander, Martin Howell, Teixeira-Pinto Armando, Carmel Hawley, Shilpa Jesudason, Amanda Walker, Fiona Mackie, Sean Kennedy, Steve McTaggart, Hugh McCarthy, Simon Carter, Siah Kim, Sam Crafter, Reginald Woodleigh, Chandana Guha, Germaine Wong

**Affiliations:** 1 Centre for Kidney Research at The Children’s Hospital at Westmead, Westmead, New South Wales Australia; 2 Sydney School of Public Health, University of Sydney, Sydney, New South Wales Australia; 3 College of Medicine and Public Health, Flinders University, Adelaide, Adelaide, South Australia; 4 Department of Renal Medicine, Royal Children’s Hospital in Melbourne, Victoria, Australia; 5 Department of Renal Medicine, Sydney Children’s Hospital, Sydney, Randwick, New South Wales Australia; 6 Department of Renal Medicine, Lady Cilento Children’s Hospital, Brisbane, Queensland Australia; 7 Department of Renal Medicine, Women’s and Children’s Hospital, Adelaide, South Australia; 8 Faculty of Medicine, Princess Alexandra Hospital Southside Clinical Unit, Queensland, Australia; 9 Department of Renal Medicine, Royal Adelaide Hospital, Adelaide, South Australia; 10 CanCare, Prostate and Breast Cancer Foundation, Surry Hills, Australia

**Keywords:** Chronic kidney disease, Dialysis, Kidney transplantation, Children, Adolescents, Patient navigator, Socioeconomic disadvantage, Health disparities, Randomised controlled trial

## Abstract

**Background:**

Chronic kidney disease (CKD) is a devastating illness associated with increased mortality, reduced quality of life, impaired growth, neurocognitive impairment and psychosocial maladjustment in children. There is growing evidence of socioeconomic disparities in health outcomes among children with CKD. Patient navigators are trained non-medical personnel who assist patients with chronic conditions journey through the continuum of care and transit across different care settings. They help vulnerable and underserved populations to better understand their diagnosis, treatment options, and available resources, guide them through complex medical systems, and help them to overcome barriers to health care access. Given the complexity and chronicity of the disease process and concerns that current models of care may not adequately support the provision of high-level care in children with CKD from socioeconomically disadvantaged backgrounds, a patient navigator program may improve the provision of care and overall health of children with CKD.

**Methods:**

The NAV-KIDS^2^ trial is a multi-centre, staggered entry, waitlisted randomised controlled trial assessing the health benefits and costs of a patient navigator program in children with CKD (stages 3–5, on dialysis, and with kidney transplants), who are of low socioeconomic backgrounds. Across 5 sites, 210 patients aged from 3 to 17 years will be randomised to immediate receipt of a patient navigator intervention for 24 weeks or waitlisting with standard care until receipt of a patient navigator at 24 weeks. The primary outcome is child self-rated health (SRH) 6-months after completion of the intervention. Other outcomes include utility-based quality of life, caregiver SRH, satisfaction with healthcare, progression of kidney dysfunction, other biomarkers, missed school days, hospitalisations and mortality. The trial also includes an economic evaluation and process evaluation, which will assess the cost-effectiveness, fidelity and barriers and enablers of implementing a patient navigator program in this setting.

**Discussion:**

This study will provide clear evidence on the effectiveness and cost-effectiveness of a new intervention aiming to improve overall health and well-being for children with CKD from socioeconomically disadvantaged backgrounds, through a high quality, well-powered clinical trial.

**Trial registration:**

Prospectively registered (12/07/2018) on the Australian New Zealand Clinical Trials Registry (ACTRN12618001152213).

## Background

CKD is a devastating illness associated with increased mortality, reduced quality of life, impaired growth, neurocognitive impairment and psychosocial problems in children. The overall annual mortality rate for children on dialysis is 35 per 1000 population and is 30-fold higher than children without CKD [1]. Such large discrepancies in mortality rates remain despite medical advances over the past two decades [1].

There is growing evidence of socioeconomic inequalities in health among children with CKD [2, 3]. Our recent work has reported that children with CKD of the lowest and second lowest socioeconomic status (SES) quartiles were at least three and two times respectively more likely to experience poorer overall health compared to the highest SES quartile [2]. Preliminary unpublished data from the Kids with Chronic Kidney Disease (KCAD) study suggest that poor health in children with CKD is not only attributed to the direct influence of the chronic illness but also reflects outcomes of the complex pathway that defines equitable access to healthcare. Socioeconomic disparities in health among children with CKD are likely attributable to a myriad of different barriers including patient-level, health system and provider factors that extend beyond biological differences [4, 5]. There are many potential barriers related to health care and may include the high costs of care, the dearth of available services that are specific to the needs of children with CKD and the lack of culturally competent care in this vulnerable population [6].

Patient navigators are trained non-medical personnel who assist patients with complex and/or chronic conditions journey through the continuum of care and transit across different care settings [7]. They help neglected and underserved populations with chronic illness to better understand their diagnoses, treatment options, and available resources, help to guide them through complex medical systems, and help them to overcome barriers to health care access and bridge gaps in transitions of care. In the context of cancer care, patient navigator programs improve patients’ satisfaction with care and treatment adherence [8]. Outside oncology, there is some evidence supporting the use of patient navigator programs in children with chronic conditions such as diabetes, asthma and obesity, to facilitate improved access to care [9]. However, the benefits of patient navigators in paediatric nephrology settings have not been assessed. There are growing concerns that the current model of care in paediatric nephrology may not adequately support the provision of high-level care for children from socioeconomically disadvantaged backgrounds. Given this, and the complexity and chronicity of the disease process, we hypothesize that a patient navigator program will lead to improvement in the provision of care and overall health of children with CKD and will be cost-effective.

The NAV-KIDS^2^ trial is a multi-centre, staggered entry, waitlisted randomised controlled trial that assesses the health benefits and costs of a patient navigator program in children with chronic kidney disease (CKD) stages 3–5, on dialysis (CKD-D) and with kidney transplants (CKD-T), who are from low socioeconomic backgrounds. The trial design and research plan are informed by extensive longitudinal observational and qualitative data from the Kids with CKD (KCAD) study [10].

### Key research question

In children with CKD (3–5), CKD-D and CKD-T and of low SES backgrounds, does a patient navigator program improve the overall health and well-being compared to standard care?

### Study objectives

#### Primary objective

To compare the self-rated health (SRH) of children with CKD randomised to the intervention (patient navigator program) arm and control (wait-listed) arm.

#### Secondary objectives

To compare the between group means of the secondary end-points including utility-based quality of life, SRH of the caregiver, caregivers’ satisfaction with healthcare, progression of kidney dysfunction, other biomarkers (kidney, liver and haematological function), the number of hospitalisations and missed school days; and compare the between group mortality rates. An additional secondary objective is to assess the cost-effectiveness of a patient navigator program in children with CKD compared to standard care, based on the incremental costs and benefits associated with implementation.

#### Tertiary objectives

To assess the fidelity, satisfaction, facilitators and barriers of a patient navigator program in children with CKD.

## Methods

### Study design

**Fig. 1:**
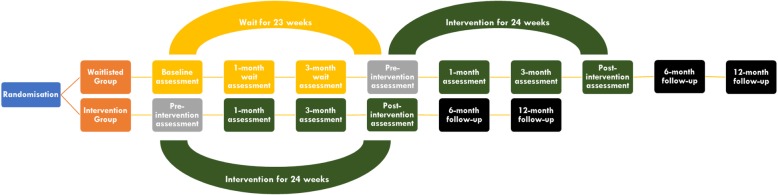
Trial schema

The NAV-KIDS^2^ trial is a multi-centre, staggered-entry, waitlisted randomised controlled trial of a patient navigator program in children with CKD. The rationale for this design is that there is some evidence of health and social benefits for patient navigator programs [9], and a general expectation that the patient navigator program will do good than harm in children with chronic illness. As such, it would be unethical to withhold the intervention from a proportion of the participants as in a traditional parallel design. The waitlisted controlled design has the benefit of allowing all eligible participants to be enrolled and receive the same intervention for the same duration of time, but with staggered entry at different time points (different waves) such that participants with delayed entry will serve as controls [11]. It will also aid recruitment as all participants will have the opportunity to access the intervention. The trial schema is shown in Fig. 1.

### Study setting

Participants will be recruited from 5 hospital sites across Australia: The Children’s Hospital at Westmead; Sydney Children’s Hospital, Randwick; Queensland Children’s Hospital; Royal Children’s Hospital Melbourne; and the Women’s and Children’s Hospital Adelaide. Recruitment will be leveraged by our observational KCAD study [10]. Currently, a total of 194 children from the KCAD study are from low SES backgrounds. Recruitment is therefore highly feasible as we will be able to fulfil around 90% of our sample size by inviting participants from the KCAD study alone. We will also recruit new participants from the five largest paediatric nephrology units in Australia.

### Eligibility criteria

#### Inclusion criteria

To be eligible for the study, participants must satisfy all of the following criteria.

Diagnosed with CKD (3–5) or CKD-D or CKD-T.

Aged 3–17 years (inclusive).

Low SES background. Low SES families are defined as those satisfying any of the following criteria:

Weekly household income of less than the median gross household income, $1250 (AUD) per week;

Poor or very poor self-perceived financial status;

Single parenting on social benefits;

Both parents are unemployed; or

Families living in public housing.

#### Exclusion criteria

Life expectancy of less than 12 months.

Unable or unwilling to provide consent by the caregiver (and child assent if the child is 16 years or over).

### Consent process

Consent will be obtained by the trial coordinator or a research assistant. For children under 16 years, a parent or caregiver will sign the consent form on behalf of the child. Children under 16 who are considered by the investigator to be of requisite maturity to understand the study requirements will be asked to assent and countersign the parent consent form. This will be determined by the investigator on a case-by-case basis and will vary with the level of maturity of the child participant. For children aged 16 or 17 years, a parent or caregiver will sign the parent consent form and the child will countersign the parent consent form. For participants who do not speak English, a phone interpreter service will be used during the consent process.

### Intervention

Navigators will be trained using a training program developed for the study and will have support from an existing patient navigator organisation (CanCare). The navigator will work with patients, caregivers, and health professionals to achieve better care and health for patients through involvement in their social, community and health organisational networks. It is a complex and individualised intervention that will be tailored to the needs of the patients and their families [12]. The navigator will follow a four-by-five matrix of tasks (identification of task categories, facilitation tasks, identification of networks, document and review) and networks (patient, provider, non-clinical staff, supportive services, medical records/electronic medical records). [13] Domains of tasks in this study are further described below:

1. *Identification of task categories for a specific patient and family*: navigating tasks may consist of identifying and mitigating barriers with patients and healthcare professionals. They may include telling (explaining where and when a renal biopsy will be done), inquiring (asking about the potential barriers, such as language barriers to attend the next appointment after the biopsy), supporting (listening to the fears about the interventions) and coaching (discussing the potential questions the patients and families may wish to ask in the next appointment).

2. *Facilitation for a specific patient and family*: the navigator may coordinate communication, seek advice from non-medical and medical staff and help to bring patients in for the appointments.

3. *Identification of networks*: the navigators will identify all potential network interactions that are relevant to the patients and their families. These may include: the health service providers, the non-clinical staff (administrators and receptionist), and other social support services such as social workers, community-based services, transportation, and the maintenance of activities and system tasks for patients.

4. *Document and review*: the navigator will record their own actions (for example: steps taken with or on behalf of the patients) and record other activities that are relevant to the navigator role.

Participants who do not speak English will be paired with a navigator who speaks a language they are comfortable speaking in or will have access to a phone interpreter service to translate their communications with the navigator.

### Randomisation and follow-up

The trial will be centrally coordinated at the Australasian Kidney Trials Network (AKTN). Individuals who meet the inclusion criteria and have given informed written consent will be randomised with equal probability to the intervention or the waitlisted controlled group, via an independent central web-based system. The randomisation sequence will be generated by a computerized random number generator at the AKTN, using a random permuted block design with randomly chosen block sizes. Randomisation will therefore be kept separate from recruitment (which will be conducted by research personnel at each site), to maintain allocation concealment. Randomisation will be stratified by CKD stage and recruitment site, and permuted blocks will be used for each stratum (combination of CKD stage and site) to achieve balance. It is anticipated that 21 children will be randomised to the intervention and waitlisted arms at each site (42 children in total for each site). Five different waves, across the five different sites, will be progressively recruited, in concordance with the school terms over the 12-month period. In the first wave (to be conducted at the Children’s Hospital at Westmead), children being randomised to the intervention arm will receive the intervention (patient navigator program) immediately after randomisation for 24 weeks. Assessments will be conducted pre-intervention, 1-month and 3-months into the intervention, immediately post-intervention, 6-months and 12-months after the intervention. Children randomised to the wait-list arm will wait for 23 weeks but will receive standard care during the ‘wait-period’. They will commence the intervention (patient navigator program) in week 24. Assessments during the ‘wait-period’ will be conducted at baseline, 1-month and 3-months after randomisation and immediately pre-intervention at 6-months. Similar to the intervention arm, the waitlist-controlled arm will receive the intervention for a period of 24 weeks. Assessments, using the same methods, will be conducted at the same follow-up time points as per the intervention arm. The other four waves (at the four other recruiting sites) will occur sequentially at the beginning of each school term (3 months apart). The schedule for assessments for each group is shown in Table 1 (immediate treatment group) and Table 2 (waitlisted group).

**Table 1:** Schedule of assessments for immediate treatment group

	Screen	Random	Treat (1mo)	Treat (3mo)	End treat (6mo)	Post-treat (6mo)	Post-treat (12mo)
Day		**0**	**28**	**84**	**168**	**336**	**504**
Eligibility Criteria	x						
Demographics		x					
CKD Information		x	x	x	x	x	x
Medical History		x	x	x	x	x	x
Physical Examination		x	x	x	x	x	x
Bloods		x			x	x	x
Immunosuppressive Medications		x	x	x	x	x	x
Concomitant Medications		x	x	x	x	x	x
SRH (Child and Caregiver)		x	x	x	x	x	x
Educational Background		x					
School Absenteeism		x	x	x	x	x	x
HUI Questionnaire		x	x	x	x	x	x
Caregiver Satisfaction Questionnaire (Caregiver)		x	x	x	x	x	x
Patient Navigator Satisfaction Questionnaire (Caregiver)			x	x	x		
Adverse events			x	x	x	x	x
Hospitalisation			x	x	x	x	x
Data linkage (NDI, ANZDATA, MBS, PBS)							x
Qualitative interviews/questionnaires (subset of participants only)		x		x	x	x	

Abbreviations*. CKD*: Chronic kidney disease, *SRH*: Self-rated health, *HUI*: Health utilities index, *NDI*: National death index, *ANZDATA*: Australia and New Zealand Dialysis and Transplant Registry, *MBS*: Medicare Benefits Schedule, *PBS*: Pharmaceutical Benefits Scheme

**Table 2:** Schedule of assessments for waitlisted group

	Screen	Random	Wait (1mo)	Wait (3mo)	Start treat	Treat (1mo)	Treat (3mo)	End treat (6mo)	Post-treat (6mo)	Post-treat (12mo)
Day		**0**	**28**	**84**	**168**	**196**	**252**	**336**	**504**	**672**
Eligibility Criteria	x									
Demographics		x								
CKD Information		x	x	x	x	x	x	x	x	x
Medical History		x	x	x	x	x	x	x	x	x
Physical Examination		x	x	x	x	x	x	x	x	x
Bloods		x			x			x	x	x
Immunosuppressive Medications		x	x	x	x	x	x	x	x	x
Concomitant Medications		x	x	x	x	x	x	x	x	x
SRH (Child and Caregiver)		x	x	x	x	x	x	x	x	x
Educational Background		x								
School Absenteeism		x	x	x	x	x	x	x	x	x
HUI Questionnaire		x	x	x	x	x	x	x	x	x
Caregiver Satisfaction Questionnaire (Caregiver)		x	x	x	x	x	x	x	x	x
Patient Navigator Satisfaction Questionnaire (Caregiver)						x	x	x		
Adverse events			x	x	x	x	x	x	x	x
Hospitalisation			x	x	x	x	x	x	x	x
Data linkage (NDI, ANZDATA, MBS, PBS)										x
Qualitative interviews/ questionnaires (subset of participants only)		x					x	x	x	

Abbreviations*. CKD*: Chronic kidney disease, *SRH*: Self-rated health, *HUI*: Health utilities index, *NDI*: National death index, *ANZDATA*: Australia and New Zealand Dialysis and Transplant Registry, *MBS*: Medicare Benefits Schedule, *PBS*: Pharmaceutical Benefits Scheme

### Outcome measures

The primary study end-point is SRH of the child 6 months after the completion of the intervention (i.e. 6-months post-treatment). SRH, a patient-reported health outcome, is a validated composite measure of global health status [14]. It is a broad and holistic measure that accommodates the World Health Organisation (WHO) defined concept of health [15]. SRH is also a stable measure of health over time. [16] Prior studies have shown that children (from age 8 onwards) can accurately communicate their health symptoms in a meaningful way and can provide valuable insights into their own health [17]. Below the age of 8 years, children may have difficulty interpreting and understanding the response categories. As such, parent-rated health of the child will be used as a proxy for children between the ages of 3 to 7 years. Parent-rated health has been validated for use in young children (as young as pre-schoolers). Recent work indicates that child self-rated health and parental ratings of the child’s overall health are associated with the child’s physical and mental health [16, 18, 19].

**Fig. 2:**
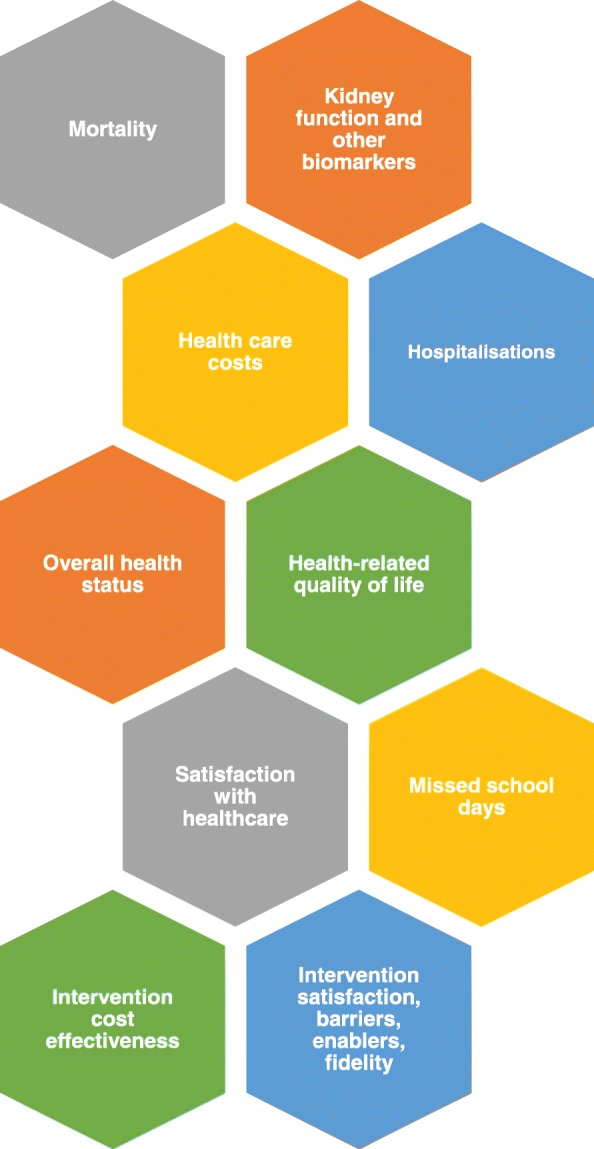
Outcome domains to be assessed

The secondary end-points are the mean differences in the SRH of the child and caregiver, utility-based quality of life (QOL) estimates, progression of kidney dysfunction calculated using the modified Schwartz creatinine-based formula [20] for the estimated glomerular filtration rate (eGFR), other biomarkers (urea, albumin, bilirubin, alanine transaminase, alkaline phosphatase, gamma glutamyl transferase, haemoglobin, white cell count, platelets), caregivers’ satisfaction with healthcare, the number of hospitalisations and missed school days, direct health-care costs and mortality rates between the intervention and the waitlisted controlled arms, up to 12-months after the completion of treatment. Serum creatinine and other biomarkers have been included to consider the impacts of the intervention on kidney, liver and haematological function, since these outcomes may be affected by changes in quality of care and adherence as a result of the intervention. All outcome domains being considered in the study are shown in Fig. 2 and all timepoints for outcome assessments are shown in Table 1 and Table 2.

The outcome assessors and data analysts will be blinded to treatment allocation, although blinding will not be feasible for the children, caregivers or clinicians and other researchers involved in study management. Utility-based QOL will be assessed using the Health Utility Index (HUI-3) [21], which will be completed by the parent for children aged 3–7 years and by the child for those aged 8 years and above. Serum creatinine and other biomarkers (urea, albumin, bilirubin, alanine transaminase, alkaline phosphatase, gamma glutamyl transferase, haemoglobin, white cell count, platelets) will be measured from blood samples. Caregiver satisfaction with the healthcare their child receives will be assessed with a questionnaire covering issues such as caregivers’ perceived access to care and their confidence in navigating the healthcare system. All-cause, cardiovascular (CV) and non-CV mortality up to 12-months after completion of treatment will be obtained using data linkage with the National Death Index, maintained by the Australian Institute and Health and Welfare (AIHW).

CKD-related outcomes up to 12-months post-treatment among those on dialysis and with kidney transplants will be obtained via data linkage (at 12 months after completion of treatment) with the Australia and New Zealand Dialysis and Transplant (ANZDATA) registry. Healthcare resource use will be estimated using parent-reported hospitalisations and linked Medicare Australia data for outpatient healthcare use (including drug costs from the Pharmaceutical Benefit Scheme (PBS) and healthcare costs from the Medicare Benefits Schedule (MBS)). Costs will be estimated by applying diagnosis-related groups (DRG) or Medicare unit costs and will also include the costs of the patient navigator program. Healthcare costs for the intervention and control groups will be calculated for the following time points: 3 months into treatment, immediately post-treatment, 6 months post-treatment and 12 months post-treatment.

### Trial infrastructure

The trial will be centrally coordinated at the Australasian Kidney Trials Network (AKTN). The trial will be overseen by a Steering Committee, consisting of investigators from the recruiting sites and two consumer representatives. The committee will have two teleconferences before study initiation and teleconferences quarterly during study recruitment and follow-up. The data safety monitoring board (DSMB), consisting of clinical, statistical as well as clinical trial experts, will have the responsibility for monitoring of adverse events and will ensure the safety of children and families is protected.

### Economic evaluation

An economic evaluation will be conducted to determine the incremental costs and benefits of a patient navigator program for improving the overall health of children with CKD compared to standard care. Initially, a within-trial economic evaluation will be conducted, which will be extended to a modelled evaluation over a longer time horizon, using a patient-level simulation model. Data sources including the utility-based QOL estimates to generate quality adjusted life years (QALYs), all other primary and secondary outcomes such as the proportion of participants reporting better overall health, and costs generated from the trial will be included in the evaluation. We will take a healthcare funder perspective. Therefore, costs will include all intervention costs and all healthcare resource use over the trial duration, including inpatient admissions, ED presentations and outpatient resource use. Both costs and benefits will be discounted at 5% per year. An incremental cost per additional patient avoiding fair/poor health, and incremental cost per QALY gained in the intervention group, compared to the wait-listed group will be calculated with results plotted on a cost-effectiveness plane. We will use a patient level microsimulation model to examine costs and outcomes over a longer time horizon. A cost-effectiveness acceptability curve will be plotted to show the probability at which an intervention is considered cost-effective, for a given willingness to pay threshold.

### Process evaluation

We will use a mixed methods approach to assess the barriers and enablers for acceptance and uptake of the program. The process evaluation will be conducted and reported in accordance with Medical Research Council guidance [22]. All participating caregivers will complete the Patient Navigator Satisfaction Questionnaire at three time points (1 month into the intervention, 3 months into the intervention and immediately post-intervention), to assess their perception of the intervention over time. In addition, qualitative semi-structured interviews and questionnaires will be conducted by trained research personnel with a purposive sample of children and family members (min. *n* = 30) prior to the intervention, 3 months into the intervention, immediately post-intervention, and 6 months post-intervention. The topics will include: acceptance of the navigator program; perceived barriers, challenges and enablers for implementation; and the perceived benefits and harms of the intervention. Key questions will also be used to assess intervention fidelity regarding perceptions of care received by the participants. Assessment of intervention fidelity across the sites will also be carried out through key-informant interviews of study staff (n = 30 interviews with navigators and healthcare professionals). Questioning will also assess barriers and enablers to program sustainability (short and long-term) and implementation of the intervention into standard care.

### Sample size calculation

The sample size was calculated for the analysis of the 5-point Likert scale of the SRH of the child (and parent-rated health for younger children) using logistic regression. Assuming a dropout rate of 20%, a total of 210 children (105 in each arm) will be required for a final sample of 166 patients. This sample size will allow us to detect an odds ratio (OR) of 2.3, with approximately 80% power and a significance level of 0.05. Data from the KCAD study indicated the OR of children from the lowest SES quartile reporting poor and fair health (compared with good, very good and excellent health) was at least 2.08 [2]. Therefore, an OR of 2.3 is a clinically significant change in SRH with the proposed intervention.

### Statistical analyses

All data will be analysed according to the intention to treat principle. The primary outcome, SRH of the child between the intervention and waitlisted groups at 6 months post-treatment, will be compared using logistic regression modelling. The second consecutive wave will also allow for a pre and post analysis. We will use robust standard errors to account for clustering within centres. In addition, we will analyse the longitudinal change in SRH up to 12-months after the intervention, using a mixed model. The degree of change in SRH over the various time points will also be examined for association with key baseline characteristics of the participants such as age, gender, CKD stage, by examining the time interaction with these variables. Secondary endpoints (continuous and count data) such as number of hospitalisations, utility-based QOL, caregiver satisfaction and number of missed school days will be analysed using Poisson regression to compare the difference between the intervention and waitlisted groups. Subgroup analyses based on the different waves (i.e. the recruiting sites) and CKD stage will also be conducted. Time to event analyses will be used to estimate the rate of death between the intervention and waitlisted groups using Cox proportional hazards model, stratified by CKD stage at the time of randomisation. A *p*-value of 0.05 will be used to indicate statistical significance.

## Discussion

Children with CKD suffer from significant physical, cognitive and psychological complications. Apart from reduced life expectancy and QOL, they are confronted with a diverse range of adverse health and social outcomes. The NAV-KIDS^2^ trial will provide clear evidence of the effectiveness and cost-effectiveness of a new intervention, through a high quality, well-powered clinical trial. It is informed by extensive observational and qualitative data from the KCAD study that highlight the current research gaps. This trial also has a number of innovative features. It is enriched by restricting the participants to those of low SES backgrounds, as prior work has shown that patient navigation interventions focused on communities-at-risk demonstrated a much greater impact of patient navigation [7]. The staggered entry, wait-listed controlled trial design not only ensures trial efficiency, but also allows adjustment for other external seasonal events, which are potential confounders to the intervention effects. As the infrastructure for the program will be developed during course of the trial, if proven effective, the navigator program can be rapidly implemented into clinical care.

## Abbreviations

AIHW: Australian Institute of Health and Welfare
AKTN: Australasian Kidney Trials Network
ANZDATA: Australian and New Zealand Dialysis and Transplant Registry
AUD: Australian dollars
CKD: chronic kidney disease
CKD-D: chronic kidney disease on dialysis
CKD-T: chronic kidney disease with a kidney transplant
CV: cardiovascular
DRG: diagnosis-related group
DSMB: data safety monitoring board
ED: emergency department
HUI-3: Health Utilities Index Mark 3
KCAD: Kids with CKD study
MBS: Medicare Benefits Schedule
OR: odds ratio
PBS: Pharmaceutical Benefits Scheme
QALY: quality-adjusted life year
QOL: quality of life
SES: socioeconomic status
SRH: self-rated health
WHO: World Health Organization
